# Effect of Ethylene and Abscisic Acid on Steroid and Triterpenoid Synthesis in *Calendula officinalis* Hairy Roots and Saponin Release to the Culture Medium

**DOI:** 10.3390/plants11030303

**Published:** 2022-01-24

**Authors:** Michał Markowski, Abdulwadood Shakir Mahmood Alsoufi, Anna Szakiel, Marek Długosz

**Affiliations:** 1 Department of Plant Biochemistry, Institute of Biochemistry, Faculty of Biology, University of Warsaw, 1 Miecznikowa Street, 02-096 Warsaw, Poland; szakal@biol.uw.edu.pl (A.S.); mdlugosz@biol.uw.edu.pl (M.D.); 2 Department of Biology, College of Science, University of Tikrit, P.O. Box 42, Tikrit 34001, Iraq; abd.alhamdany@yahoo.com

**Keywords:** abscisic acid, ethylene, hairy roots, marigold *Calendula officinalis* L., plant in vitro cultures, saponins, sterols, triterpenoids

## Abstract

Phytohormones (plant growth regulators) can be applied as efficient elicitors to enhance the productivity of plant in vitro cultures, due to their significance in regulating the plant metabolism and strong influence on plant defense responses. In the present study, the effects of exogenous ethylene (ETY, applied in the form of ethephon as an ethylene-generating agent) and abscisic acid (ABA) on the synthesis of triterpenoids and steroids in *Calendula officinalis* hairy roots were investigated. ABA appeared to be an efficient elicitor of the biosynthesis of triterpenoid oleanolic acid (almost two-fold) and the release of its glycosides (saponins) to the culture medium (up to 6.6-fold). ETY had only a slight effect on triterpenoid metabolism; instead, it strongly influenced steroid metabolism, leading to profound modifications of the quantitative profiles of these compounds, particularly the ratio of stigmasterol to sitosterol. Both the applied phytohormones influenced the interplay between steroid and triterpenoid biosynthetic pathways, revealing the symptoms of their competition.

## 1. Introduction

Hairy root cultures (HRCs) are differentiated cultures of transformed roots which are induced by the infection with bacterium *Rhizobium rhizogenes*, also known as *Agrobacterium rhizogenes* [1,2,3]. HRCs are plant in vitro cultures that are particularly popular in biotechnology due to their numerous advantages, e.g., high growth rates without an external supply of phytohormones, genetic and biochemical stability, ease of maintenance, and the ability to synthesize a wide range of valuable phytochemicals with various biological and pharmacological activities [4,5]. However, the productivity of HRCs is not always sufficient for the economic viability, and several biotechnological strategies have been developed for its improvement. This includes screening and selection of high-yield lines, optimization of culture media and culture conditions, replenishment of nutrients and precursor feeding, in situ product removal to overcome feedback inhibition, genetic engineering, as well as application of phytohormones and other elicitors [6,7]. Generally, elicitors mimic the effects of stresses and thereby activate the specific biosynthetic pathways, which results in the increased contents of specialized metabolites. Elicitation is currently one of the most practically feasible and most effective strategies for enhancing the production of phytochemicals in plant biotechnology [8,9,10,11,12]. In this context, HRCs can serve as a suitable model for the investigation of the influence of different biotic and abiotic elicitors [13,14].

Among biotic elicitors, phytohormones (plant growth regulators) are particularly of interest due to their evident significance in natural processes regulating the plant metabolism and their strong influence on plant defense responses [15]. Therefore, well-known substances, such as jasmonic acid (JA), methyl jasmonate, and salicylic acid, are commonly applied as elicitors in various plant in vitro cultures, including HRCs [13,16,17]. However, other phytohormones, e.g., abscisic acid (ABA) or ethylene (ETY), can also significantly influence the plant metabolism and hence modify the productivity of desired phytochemicals.

ETY is regarded as a multifunctional phytohormone that regulates diverse processes in plant growth, development, and stress responses throughout the plant life cycle, including seed germination, root development, shoot and root growth, formation of adventitious roots, abscission of leaves and fruits, flowering, sex determination, and senescence of flowers and leaves. ETY also mediates adaptive responses to a variety of stresses, such as drought, flooding, pathogen attack, and high salinity [18,19]. Another phytohormone, ABA, also plays an essential role in multiple physiological processes of plant growth and development, such as stomatal closure, cuticular wax accumulation, leaf senescence, bud dormancy, seed maturation, osmotic regulation, and growth inhibition. ABA controls downstream responses to abiotic and biotic environmental changes through both transcriptional and posttranscriptional mechanisms, playing an important role in mitigating various stress responses [20]. Thus, both phytohormones are involved in mechanisms of plant response to various stress factors, and they are capable of modifying metabolic pathways leading to defense phytochemicals.

The objective of the present investigation was to examine the effectiveness of ETY and ABA applied as elicitors in *Calendula officinalis* L. HRCs producing triterpenoid saponins. Triterpenoids are a group of compounds with a large variety of structures (usually based on the 4- or 5-ring carbon skeleton of various arrangements, often occurring in the form of glycosides, referred to as saponins) and functions. Their production in plant in vitro cultures, including hairy roots, is an important field of investigation in biotechnology. Triterpenoids are the compounds originating from squalene cyclization, parallel to sterols, i.e., tetracyclic compounds based on perhydro-1,2-cyclopentano- phenantren moiety. Sterols are integral components of the membrane lipid bilayer and they are involved in the maintenance of membrane homeostasis, whereas triterpenoids are believed to play an important role in plant chemical defense and interactions with the environment. Sterols and triterpenoids distinctly differ in their functions, and therefore, they are commonly regarded as general and specialized metabolites, respectively [21]. Since both sterols and triterpenoids are synthesized as products of one common precursor, 2,3-oxidosqualene, the trials of stimulation of triterpenoid biosynthesis in plants and plant in vitro cultures including hairy roots often concern the problem of the possible competition [22].

*Calendula officinalis* hairy roots, induced by transformation with the wild-type *Rhizobium rhizogenes* strain ATCC 15834, produce bioactive triterpenoid saponins, i.e., glycosides of oleanolic acid (OA), and excrete these compounds into the culture medium [23]. The previous studies revealed that this culture can serve as a suitable research model for investigations on various aspects of productivity improvement, e.g., optimization of culture media [24] or elicitation with various abiotic and biotic factors [13,14,25]. Apart from the main goal of the study, i.e., the assessment of the possible intensification of triterpenoid production, the performed studies involved the determination of steroid content, which allowed the monitoring of the relationship between triterpenoid and steroid pathways, parallel after squalene cyclization. The phytohormones applied in the present study, ETYand ABA, were supposed to interfere with primary (general) and secondary (specialized) metabolisms; therefore, the symptoms of the possible competition between the investigated pathways could be expected.

## 2. Results

### 2.1. The Identification of Triterpenoids and Steroids in C. officinalis HRCs

**Figure 1:**
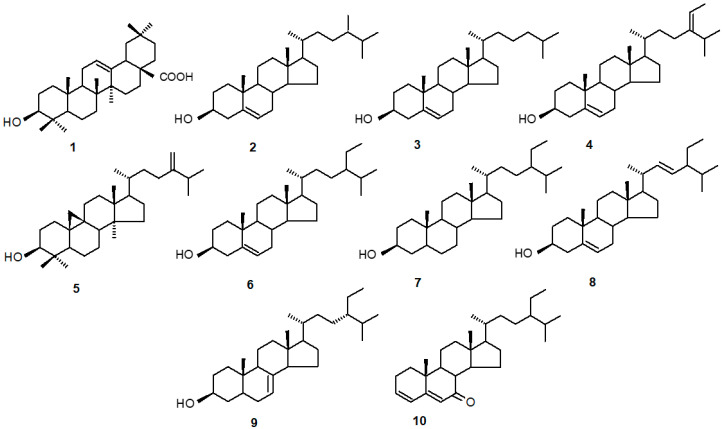
The structures of triterpenoid (oleanolic acid) and steroids identified in *Calendula officinalis* hairy roots. (1) oleanolic acid; (2) campesterol; (3) cholesterol; (4) isofucosterol; (5) 24-methylenecycloartanol; (6) sitosterol; (7) sitostanol; (8) stigmasterol; (9) stigmast-7-en-3-ol; (10) tremulone.

*C. officinalis* HRCs applied in the present study were shown to be capable of synthesizing one triterpenoid acid, i.e.,oleanolic acid, OA (3β-hydroxy-olean-12-en-28-oic acid), found both in the free form (accumulated in the hairy root tissue) as well as its glycosides, which can be accumulated in the tissue but also released to the surrounding medium. The identification of OA was confirmed by the GC-MS (gas chromatography-mass spectrometry) method, according to the MS spectrum, as well as the comparison of the retention time and chromatographic mobility with the authentic standard (Section 4.6). The content of saponins was determined as the amount of their aglycone released after acid hydrolysis (Section 4.5). The analysis of the GC-MS data of free steroids fraction revealed the occurrence of nine compounds. Their identifications were made on the basis of the MS spectra, retention time, and chromatographic mobility of available authentic standards, as well as the comparison with the data from MS libraries and the literature (Table S1). Free steroids can be unequivocally identified without derivatization by their fairly consistent mass spectra, in which a molecular ion (usually of significant intensity) was easily observed, along with other diagnostic ions produced after the loss of 3β-hydroxy group as water [M-18]^+^ and subsequent fragmentation by a retro-Diels-Alder reaction [26,27,28]. Three compounds were most prevalent in all samples: stigmasterol (22*E*-stigmasta-5,22-dien-3β-ol), sitosterol (stigmast-5-en-3β-ol), and campesterol (24*R*-ergost-5-en-3β-ol), which are typical and very common plant sterols. Other three sterols including cholesterol (cholest-5-en-3β-ol), isofucosterol (24*Z*-stigmasta-5,24(28)- dien-3β-ol; synonym: Δ5-avenasterol), and stigmast-7-en-3-ol were less prevalent. The fraction also contained three steroid compounds, i.e., sitostanol (5α-stigmastan-3β-ol, saturated sitosterol), ketone tremulone (stigmasta-3,5-dien-7-one), and 24-methylenecycloartanol (9β-24-methylene-9,19-cyclolanostane, intermediate in sterol biosynthesis). The structures of all identified compounds are presented in Figure 1.

### 2.2. The Influence of ETYon the C. officinalis Hairy Root Growth and Productivity

**Figure 2:**
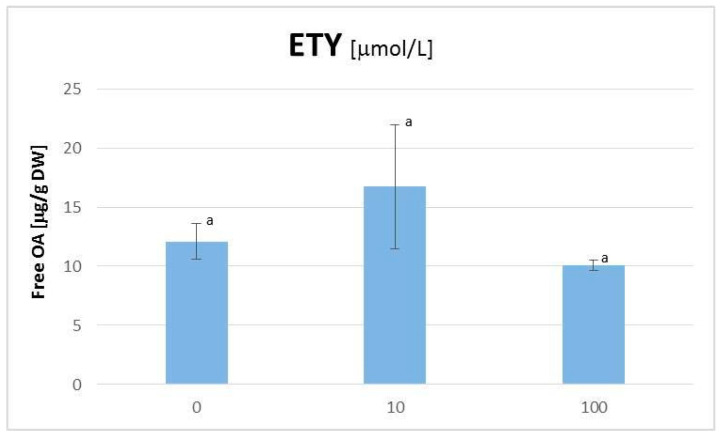
The content of free oleanolic acid in *Calendula officinalis* hairy root tissue after elicitation with ethylene. Bars sharing a common letter are not significantly different (*p* < 0.05).

**Figure 3:**
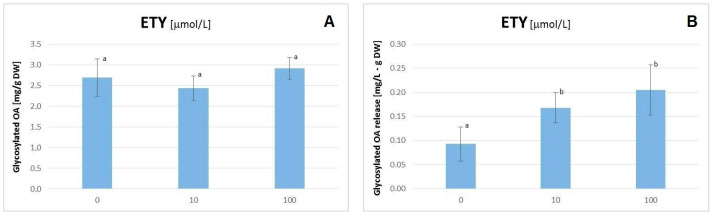
The content of oleanolic acid in the form of glycosides accumulated in hairy roots (**A**) and released to the medium (**B**) after elicitation with ethylene. Bars which do not share a common letter are significantly different (*p* < 0.05).

The elicitation with ETY, applied in the form of ethephon as an ETY-generating agent, did not influence significantly the *C. officinalis* hairy root growth; i.e., neither fresh mass nor dry weight was changed in a statistically significant manner (Figures S1 and S2). Likewise, the influence of the ETY treatment on triterpenoid metabolism was also rather slight; free OA accumulation in hairy root tissue was moderately increased at the concentration of 10 μM ETY and decreased at the concentration of 100 μM ETY (however, the results were not statistically significant according to the considerable scattering of results; Figure 2).The latter effect was accompanied by more than two-fold increase of saponin release to the medium (the results were statistically significant; Figure 3B). Simultaneously, ETY had practically no influence on the synthesis and accumulation of OA glycosides in hairy root tissue (Figure 3A).

The effect of elicitation with ETY on the steroid synthesis (Table 1) seemed to be opposite to that exerted on OA. At the concentration of 10 μM ETY, a 22% decrease of the total steroid content was observed, whereas it slightly (by 5%) increased at 100 μM ETY. The modifications of the content of the two main sterols, stigmasterol and sitosterol, were particularly significant. At the lower ETY concentration, the content of both stigmasterol and sitosterol decreased by 13% and 2-fold, respectively (the results were statistically significant only for sitosterol). At the higher ETY concentration, the content of stigmasterol increased by 20%, whereas the sitosterol content decreased by two-fold, as in the case of elicitation with 10 μM ETY (all results were statistically significant). Thus, the ratio of stigmasterol to sitosterol, which accounted for approximately 3:1 in non-elicited roots, was enhanced to 6:1 in roots elicited with the low ETY concentration and up to 8:1 in the roots elicited with the high ETY concentration.

The contents of campesterol and isofucosterol decreased after the application of the lower ETY concentration (the results were statistically significant for isofucosterol) and increased at the high ETY concentration. Thus, these changes were parallel to those exerted on stigmasterol. In turn, the content of cholesterol increased statistically significantly more than two-fold after the elicitation of both ETY concentrations, whereas the contents of stigmast-7-en-3-ol, sitostanol, and tremulone decreased (the results were statistically significant in the case of stigmast-7-en-3-ol and tremulone at the higher ETY concentration; the results for sitostanol were not statistically significant). The content of 24-methylenecycloartanol, one of the sterol precursors, stayed unchanged after treatment with the lower ETY concentration and decreased statistically significantly by 38% in hairy roots elicited by 100 μM ETY.

### 2.3. The Influence of ABA on the C. officinalis Hairy Root Growth and Productivity

**Figure 4:**
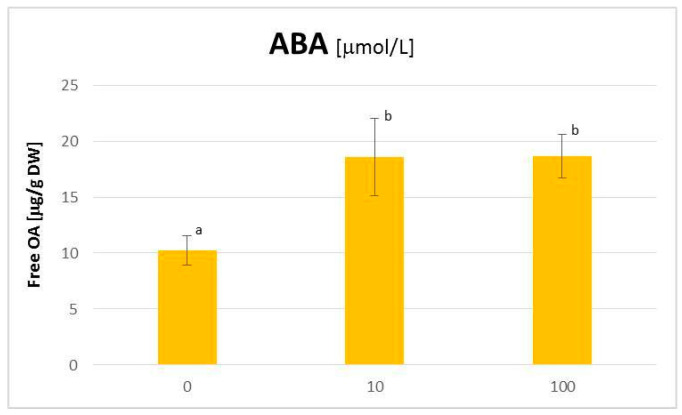
The content of free oleanolic acid in *Calendula officinalis* hairy root tissue after elicitation with abscisic acid. Bars which do not share a common letter are significantly different ( *p*< 0.05).

**Figure 5:**
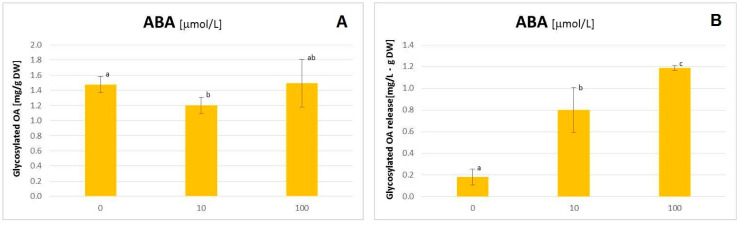
The content of oleanolic acid in the form of glycosides accumulated in hairy roots (**A**) and released to the medium (**B**) after elicitation with abscisic acid. Bars which do not share a common letter are significantly different (*p* < 0.05).

The applied concentrations of ABA influenced the hairy root growth slightly negatively; both the fresh mass and the dry weight were decreased by approx. 30% at a concentration of 10 μmol/L (Figures S3 and S4). Simultaneously, elicitation with ABA exerted a 1.8-fold increase (statistically significant) of the content of free OA the hairy root tissue (Figure 4), whereas no enhanced accumulation of saponins was observed (Figure 5A). Instead, the intensive release of these compounds to the medium was noticed; it increased 4.4- and 6.6-fold as compared to the control at concentrations of 10 and 100 µmol/L ABA, respectively (all obtained results were statistically significant; Figure 5B).

The treatment with both concentrations of ABA diminished the total content of steroids by approximately 15% (Table 2). The most affected compound was isofucosterol, with 2- and 3.5-fold decreases at 10 and 100 µmol/L ABA concentrations, respectively (however, the results were statistically significant only at the higher ABA concentration). The contents of the major sterols, sitosterol, stigmasterol, and campesterol, were diminished by approximately 20% (however, the results were not statistically significant for sitosterol). In contrast, the contents of cholesterol and 24-methylenecycloartanol were enhanced, both approximately two-fold and statistically significantly, as well as the contents of stigmast-7-en-3-ol and sitostanol, which increased by approximately 30% (however, the results were statistically significant only for sitostanol at the lower ABA concentration). The content of tremulone increased more than two-fold in hairy roots elicited with the lower ABA concentration, whereas it decreased by almost 60% in the roots applied to the higher ABA concentration treatment (all changes were statistically significant).

## 3. Discussion

Phytohormones are a group of crucial signal molecules regulating all aspects of plant growth and development and thus involved in modifying the interplay between the plant general and specialized metabolisms. Therefore, many phytohormones have been widely used as efficient elicitors of the productivity of plant in vitro cultures [9,10,11,12,29]. In the present study, the effects of exogenous ETY(applied in the form of ethephon, which acted as an ETY-generating agent) and ABA on the synthesis of triterpenoids and steroids in *C. officinalis* hairy roots were investigated. Both phytohormones are generally considered as involved in the stress maintenance and processes of maturation (or even senescence) rather than in intensive plant growth. Therefore, it could be expected that they would stimulate the biosynthesis of specialized metabolites, i.e., triterpenoids and triterpenoid saponins, more efficiently than biosynthesis of general metabolites as phytosterols, required as the membrane constituents in dividing cells. However, phytosterols and derived steroids are also involved in plant response to stress (e.g., as membrane fluidity and permeability modulators) [30]; therefore, the final results of the influences of these phytohormones on the general and specialized metabolism trade-off cannot be precisely predicted.

The results of the present study revealed that ETY exerted a rather slight influence on triterpenoid metabolism, depending on the concentration of the applied elicitor. At high ETY concentration, the decrease of free OA accumulation was observed, accompanied by a two-fold increase of saponin biosynthesis and their subsequent immediate release to the medium. Simultaneously, ETY significantly influenced the metabolism of steroids, which resulted in profound modifications of the profiles of these compounds, particularly the characteristic ratio of stigmasterol to sitosterol. This ratio is often changed during a metabolic reaction to biotic and abiotic stresses [30,31]. Another noticeable effect was the decrease of the content of 24-methylenecycloartanol at high ETY concentrations, pointing to the inhibition of phytosterol biosynthesis, possibly at the level of squalene cyclization.

The influence of ABA treatment on the triterpenoid productivity of *C. officinalis* hairy roots was much stronger than that of ETY treatment. The significant increase of the biosynthesis of OA (almost twice) and its glycosides released to the medium (up to 6.6-fold) was observed. Simultaneously, the decrease of the steroid content (by 15%) was noticed; however, the modifications of the profiles of these compounds were different than in the case of ETY, that is, no influence of stigmasterol:sitosterol ratio was exerted by ABA; instead, the most affected compounds were less abundant compounds i.e., isofucosterol and cholesterol. Interestingly, the decrease of the total content of steroids was accompanied by a two-fold increase of 24-methylenecycloartanol. This might suggest that ABA, in contrast to ETY, inhibited phytosterol biosynthesis after squalene cyclization.

ETY (applied in the form of ethephon) has been used in various trials of the enhancement of productivity of plant in vitro cultures, e.g., the biosynthesis of diosgenin in *Dioscorea floribunda* Mart. and Gal. cell aggregates [32], ginsenosides in ginseng (*Panax ginseng* C.A. Meyer) adventitious root cultures [33], anthocyanins in grapevine (*Vitis vinifera* L.) suspension culture [34], or phenolic acids in *Salvia* *miltiorrhiza* Bunge hairy roots [29]. The reported effects varied, depending on plant species as well as the elicitor concentration and the time of exposure to elicitation. For example, anthocyanin biosynthesis was increased by ETY in *Vitis vinifera* suspension culture by approximately 60% after seven days of elicitation, and afterwards, this effect decreased to approximately 5% after 18 days [34]. The content of ginsenosides in ginseng adventitious root cultures increased slightly after treatment with 50 µM ETY, whereas it was inhibited at 100 µM ETY [33]. In contrast, ETY was proved to be very efficient elicitor of diosgenin in cultured cell aggregates of *Dioscorea floribunda*, increasing its content up to 72-fold [32].

ABA has been reported as an efficient elicitor of triterpenoid metabolism in plants. For example, ABA treatment of leaves of Chinese liquorice *Glycyrrhiza uralensis* Fisher resulted in a significant (by approximately80%) increase of accumulation of valuable triterpenoid saponin (i.e., glycyrrhizin) in liquorice roots [35]. In another study, ABA was applied in conditions of water deficiency and enhanced UVB radiation to improve the acclimation of grapevine (*Vitis vinifera*) to cultivation at high heights, and the treatment resulted in more than six-fold increased accumulation of squalene—the precursor of triterpenoids [36]. In plant in vitro cultures, ABA is used to enhance the production of phenolic acids in *Salvia* *miltiorrhiza* Bunge [29]; however, more often, it has been reported as a factor applied in various tissue culture systems to promote somatic embryogenesis, enhance somatic embryo quality or promote root system development [37,38]. The results obtained in the present study indicated that ABA can act as an efficient enhancer of the triterpenoid productivity in plant in vitro cultures. Nevertheless, the stimulating effect of ABA observed in this study was weaker than the enhancement of triterpenoid saponin biosynthesis and their release to the medium exerted by JA, reported previously [13,39]. JA treatment resulted in a significant increase of saponin accumulation in hairy root tissue (up to 20-fold) and their release to the medium (up to 113-fold) [13]. It can be assumed that both JA and ABA could be applied together to achieve the highest synergistic effect. However, in the case of JA treatment, a very sharp decrease in the content of sterols (approximately 60%) and a reduction of the hairy root mass were observed. Therefore, ABA elicitation seems to be less harmful for hairy root growth, and it can be preferentially applied when the inhibition of culture growth and biomass is disadvantageous.

The results of this study have widened the knowledge on the effect of biotic elicitors, particularly selected phytohormones, on the biosynthesis of triterpenoid saponins in HRCs. Saponin production in plant in vitro cultures is currently a subject of intensive research [40,41]. However, to avoid any generalizations, it should be emphasized that the final effect of any elicitation strongly depends on various factors as the plant species, the type of culture, elicitor specificity, elicitor concentration, duration of exposure, and timing of elicitor addition.

## 4. Materials and Methods

### 4.1. Plant Material

HRC line CC16 (derived from cotyledon explant) was obtained according to a previously described procedure [23]. The roots were cultivated in a ½ Murashige–Skoog liquid medium, at 23–25 °C, in the darkness on a rotatory shaker at 120 rpm. Subcultures were performed every 3–4 weeks by transferring the 1–2 cm pieces of the young branched root to 100 mL of a fresh medium.

### 4.2. Elicitation of HRCs

Freshly subcultured roots were incubated for 15–23 days to obtain at least 1.5 g of fresh weight. Afterwards, they were weighed and transferred to a fresh medium five days prior to elicitation. Appropriately weighed samples of ethephon (2-chloroethylphosphonic acid; Sigma-Aldrich, St. Louis, MO, USA) and ABA (DuchefaBiochemie, Netherlands) were used to prepare stock solutions of 0.1 mol/L in distilled water and 96% ethanol, respectively. Further, ABA solutions were prepared in distilled water. The solutions were sterilized by filtration through a 0.22 m syringe filter (Millipore, Bionovo, Legnica, Poland) and added to the culture medium to obtain the final concentrations of 10 and 100 µmol/L. The control cultures (in ABA treatment experiments) were treated with an adequate volume of 96% ethanol. The incubation period before harvesting the roots and the medium lasted five days.

### 4.3. Extraction of the Hairy Roots and the Culture Medium

After five days of elicitation, the culture media were filtered from the hairy roots. The harvested hairy roots were dried at room temperature for at least two weeks, whereas the culture media were directly extracted 3 times with 40 mL portions of n-butanol. Dried hairy roots were powdered and extracted using a Soxhlet apparatus for 8 h with diethyl ether and then 8 h with methanol. The obtained extracts were evaporated to dryness under reduced pressure on a rotary evaporator.

### 4.4. Fractionation of Diethyl Ether Extracts

Evaporated diethyl ether extracts were fractionated by adsorption preparative TLC (thin-layer chromatography) on 20 cm × 20 cm glass plates, manually coated with silica gel 60H (Merck, Darmstadt, Germany). A solvent system with a chloroform:methanol (97/3,*v*/*v*) mixture was used to develop the plates. Two fractions were obtained as described earlier [13,14], i.e., oleanolic acid (OA) and free steroids. The steroid fraction was directly analyzed using GC-MS (Agilent Technologies 7890A, Section 4.6), while the OA fraction was methylated with diazomethane [13,14,28].

### 4.5. Hydrolysis of Methanol and n-Butanol Extracts

Methanol extracts from roots and n-butanol extracts from the culture medium were hydrolyzed by 11% HCl in 70% methanol for 2h on a heating mantle under reflux as described earlier [25]. Subsequently, the hydrolysates were diluted with distilled water, methanol was evaporated in a rotary evaporator, and the obtained aqueous remaining were extracted 3 times with 40 mL portions of diethyl ether in a separation funnel. The obtained extract was washed with distilled water 3 times and evaporated to dryness.

The dried extracts were fractionated by preparative TLC on 20 cm × 20 cm glass plates, manually coated with silica gel 60H (Merck, Darmstadt, Germany). A solvent system with a chloroform:methanol (95/5,*v*/*v*) mixture was used for developing the plates. The obtained OA was methylated with diazomethane [13,14,28].

### 4.6. Quantification of OA by Gas Chromatography

Analyses of methylated OA were performed by gas chromatography–liquid chromatography on a Shimadzu GC-2014 instrument equipped with a flame ionization detector and a ZB-1 30 m × 0.25 mm × 0.25 μm column (Phenomenex, SHIM-POL, Izabelin, Poland). The employed parameters were: column temperature 270 °C; split 1:5; the injector and the detector temperature 290 °C.; the carrier gas (nitrogen) 1.2 mL/min. Peak identification and quantification of OA were based on a calibration curve prepared with an authentic standard of methylated OA [13,14].

### 4.7. Identification and Quantification of Steroids by Gas Chromatography–Mass Spectrometry

Wiley 9th ED. and NIST 2008 Lib. SW Version 2010 were used in GC-MS data analysis. Individual compounds were identified by comparing their mass spectra with library data and/or their chromatographic mobility and corresponding mass spectra with those of authentic standards. Analyses were performed with the use of an Agilent Technologies 7890A gas chromatograph (GC-MS; Perlan Technologies, Warszawa, Poland). The system was equipped with a 5975C mass selective detector, a G4513A autosampler, and a 30 m × 0.25 mm i.d. (inner diameter), 0.25 μm, HP-5MS UI column (Agilent Technologies, Santa Clara, CA, USA). The temperature program was applied as following: the start at 160 °C (2 min), an increase to 280 °C at 5 °C/min, and the final temperature of 280 °C held for 44 min. The other employed parameters were as follows: the carrier gas flow rate (helium), 1 mL/min; the inlet and FID (flame ionization detector) temperatures, 290 °C; the quadrupole temperature, 150 °C; the ion source temperature, 230 °C; the EI (electron ionization) energy, 70 eV; scan range (*m*/*z*), 33–500; the MS transfer line temperature, 275 °C; the FID gas (hydrogen) flow rate, 30 mL/min; the air flow rate, 400 mL/min.

The quantitation of steroids was performed using an external standard method based on calibration curves determined for an authentic standard of stigmasterol. Calibration curves were drawn between the peaks areas versus the concentration of stigmasterol (y = 35,900x − 550) in the range of 0.002–2.0 mg/mL. The correlation coefficient (r^2^) value was 0.998, the RSD (relative standard deviation) values of peak areas were less than 4%, the limit of quantification (LOQ) was attained for 3.2 μg/mL, and the limit of detection (LOD) was 1.0 μg/mL (determined by the analysis of samples with the decreasing concentration of the analyte) [27].

### 4.8. Statistical Analysis of Data

All experiments were performed in three replicates. Data are presented as the means ± the standard errors of three independent samples. For statistical analysis t-Student’s test was applied using Microsoft Excel by Microsoft, Redmont, WA, USA, and STATISTICA by TIBCO Software Inc., Palo Alto, CA, USA.

## 5. Conclusions

The results obtained in the present study revealed that the selected phytohormones, i.e., ETY and ABA, supplied as exogenous elicitors, exerted diverse effects on triterpenoid and steroid metabolism in *C. officinalis* HRCs. ABA appeared to be an efficient elicitor of the biosynthesis of triterpenoid saponins and their release to the culture medium (up to 6.6-fold). In contrast, ETY, applied in a form of ethephon, had no significant effect on triterpenoid metabolism. Instead, it strongly influenced steroid metabolism, leading to profound modifications of the quantitative profiles of these compounds, particularly the ratio of stigmasterol to sitosterol.

Both the applied phytohormones influenced the interplay between general (steroids) and specialized (triterpenoids) biosynthetic pathways, revealing the symptoms of their competition, i.e., the synthesis of triterpenoids was increased at the expense of steroids. However, the mechanism of the inhibition of steroid synthesis seems to be different; regarding the content of the steroid precursor, 24-methylenecycloartanol, ETY typically inhibited squalene cyclization, whereas ABA acted in a later step of the steroid pathway.

## Figures and Tables

**Table 1:** The content of steroids in *Calendula officinalis* hairy root cultures after elicitation with ethylene.

Compound	ETY Concentration (µmol/L)		
	0	10	100
campesterol	52.6 ± 1.6 a	41.3 ± 7.3 a	58.1 ± 3.0 a
cholesterol	2.9 ± 0.4 a	6.1 ± 0.9 b	7.2 ± 0.5 b
isofucosterol	6.7 ± 0.8 a	3.3 ± 1.1 b	7.8 ± 0.7 a
24-methylenecycloartanol	11.4 ± 2.5 a	11.5 ± 1.9 a	7.1 ± 0.7 b
sitosterol	144.0 ± 25.4 a	70.7 ± 3.1 b	71.7 ± 7.9 b
sitostanol	15.6 ± 2.9 a	10.7 ± 1.4 a	9.5 ± 0.8 a
stigmasterol	460.3 ± 4.2 a	398.9 ± 53.4 a	581.8 ± 21.9 b
stigmast-7-en-3-ol	8.1 ± 2.5 a	3.0 ± 0.7 b	2.8 ± 0.2 b
tremulone	13.2 ± 3.3 a	9.7 ± 2.4 a	2.1 ± 0.6 b
Total	714.7	555.2	748.1

Results are referenced to the hairy root dry weight and expressed as the means ± SE (standard error) of three independent samples. The means in a line which do not share a common letter are significantly different (*p* < 0.05).

**Table 2:** The content of steroids in *Calendula officinalis* hairy root culture after elicitation with abscisic acid.

Compound	ABA Concentration (µmol/L)		
	0	10	100
campesterol	43.8 ± 1.1 a	33.6 ± 3.2 b	35.5 ± 2.0 b
cholesterol	4.0 ± 0.4 a	8.3 ± 2.6 b	7.8 ± 2.1 b
isofucosterol	9.8 ± 1.4 a	5.0 ± 2.9 a	2.8 ± 1.8 b
24-methylenecycloartanol	12.4 ± 1.1 a	19.8 ± 1.9 b	24.4 ± 2.7 b
sitosterol	119.4 ± 13.8 a	103.1 ± 8.9 a	99.3 ± 20.6 a
sitostanol	18.4 ± 1.4 a	27.5 ± 1.3 b	25.4 ± 8.8 a
stigmasterol	460.9 ± 13.4 a	355.5 ± 29.3 b	370.7 ± 18.4 b
stigmast-7-en-3-ol	7.9 ± 2.0 a	8.6 ± 3.1 a	11.0 ± 4.3 a
tremulone	15.3 ± 5.9 a	34.8 ± 4.5 b	6.4 ± 2.4 c
Total	691.9	596.1	583.4

Results are referenced to the hairy root dry weight and expressed as the means ± SEs of three independent samples. The means in a line which do not share a common letter are significantly different (*p* < 0.05).

## Data Availability

Not applicable.

## Supplementary Materials

The following supporting information can be downloaded at: https://www.mdpi.com/article/10.3390/plants11030303/s1, Table S1. GC-MS (gas-chromatography-mass spectrometry) data (retention times and characteristic ions of mass spectra) of the identified steroids; Figure S1. Fresh weight of the *C. officinalis* hairy root culture after elicitation with ethylene; Figure S2. Dry weight of the *C. officinalis* hairy root culture after elicitation with ethylene; Figure S3. Fresh weight of the *C. officinalis* hairy root culture after elicitation with abscisic acid; Figure S4. Dry weight of the *C. officinalis* hairy root culture after elicitation with abscisic acid.
