# Supplementary material for: Effect of Ethylene and Abscisic Acid on Steroid and Triterpenoid Synthesis in Calendula officinalis Hairy Roots and Saponin Release to the Culture Medium
Source: Plants (Basel). 2022 Jan 24;11(3):303. doi: 10.3390/plants11030303 (PMC8839607; doi:10.3390/plants11030303)
Supplement: Supplementary file 1 [file plants-11-00303-s001.zip › plants-1560371-supplementary.pdf]

Effect of Ethylene and Abscisic Acid on Steroid and Triterpenoid Synthesis in *Calendula officinalis* Hairy Roots and Saponin Release to the Culture Medium

Table S1. GC-MS data (retention times and characteristic ions of mass spectra) of identified steroids.

Compound	Formula	Molecular weight	Retention time [min]	Mass spectrum *m/z* (relative intensity)
cholesterol	$C_{27}H_{46}O$	386,65	31,9	386 (26), 107 (50), 105 (48), 91 (57), 81 (54), 79 (46), 69 (47), 57 (87), 55 (73), 43 (100), 41 (55)
campesterol	$C_{28}H_{48}O$	400,68	34,4	400 (30), 107 (51), 105 (55), 95 (49), 83 (45), 81 (64), 71 (62), 57 (77), 55 (77), 43 (100), 41 (52)
stigmasterol	$C_{29}H_{48}O$	412.69	35,6	412 (36), 145 (64), 107 (52), 95 (100), 83 (66), 81 (90), 78 (60), 69 (67), 67 (85), 55 (69)
sitosterol	$C_{29}H_{50}O$	414.71	37,5	414 (29), 145 (54), 107 (59), 105 (60), 95 (54), 91 (49), 81 (57), 57 (68), 55 (70), 43 (100)
sitostanol	$C_{29}H_{52}O$	416.72	37,6	416 (31), 215 (82), 109 (58), 107 (83), 95 (81), 93 (64), 81 (84), 69 (60), 57 (64), 55 (81), 43 (100)
isofucosterol	$C_{29}H_{48}O$	412.37	37,8	412 (5), 314 (100), 105 (47), 95 (50), 91 (42), 83 (40), 81 (51), 69 (61), 55 (96), 43 (49)
stigmast-7-en-3-ol	$C_{29}H_{50}O$	414,70	39,2	412 (5), 314 (100), 105 (47), 95 (50), 91 (42), 83 (40), 81 (51), 69 (61), 55 (96), 43 (49)
tremulone	$C_{29}H_{46}O$	410,70	40,8	410 (32), 187 (27), 174 (100), 161 (37), 159 (26), 91 (28), 57 (28), 55 (37), 43 (44), 41 (28)
24-methylene-cycloartanol	$C_{31}H_{52}O$	440,70	42,3	440 (5), 121 (60), 119 (55), 109 (62), 107 (76), 105 (57), 95 (98), 93 (64), 81 (72), 69 (99), 55 (100)

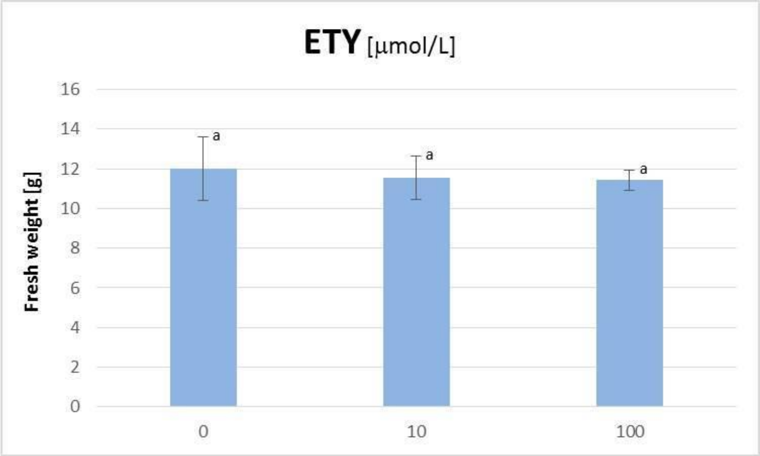

Figure S1. Fresh weight of *C. officinalis* hairy root culture after elicitation with ethylene.

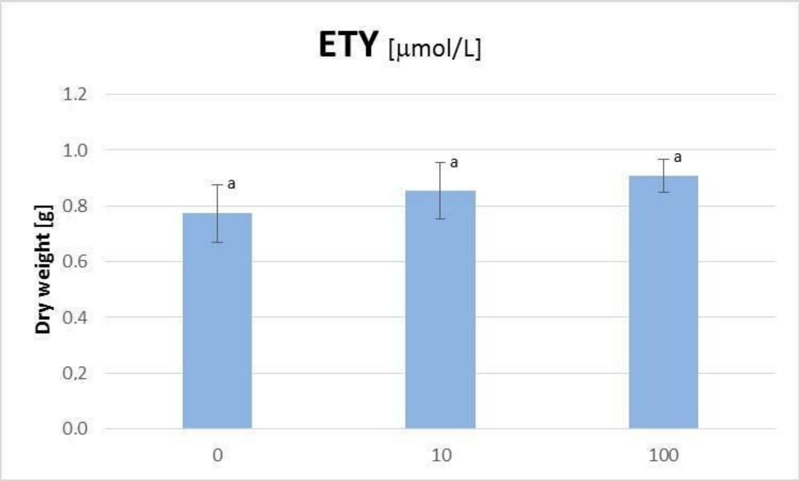

Figure S2. Dry weight of *C. officinalis* hairy root culture after elicitation with ethylene.

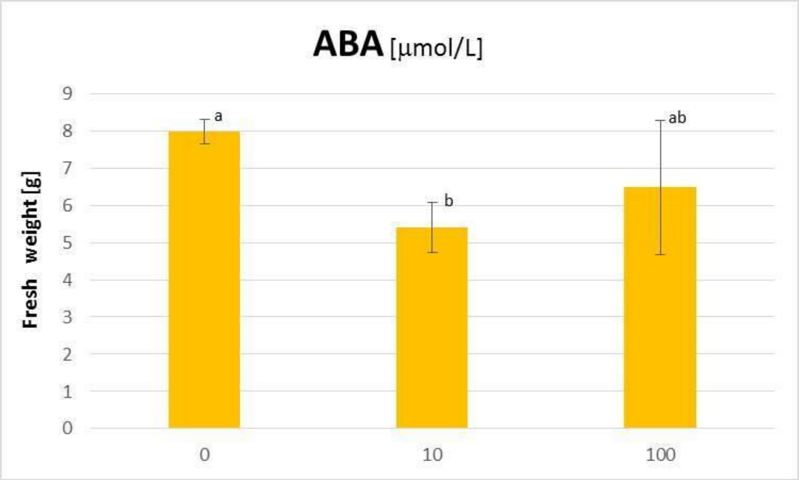

Figure S3. Fresh weight of *C. officinalis* hairy root culture after elicitation with abscisic acid.

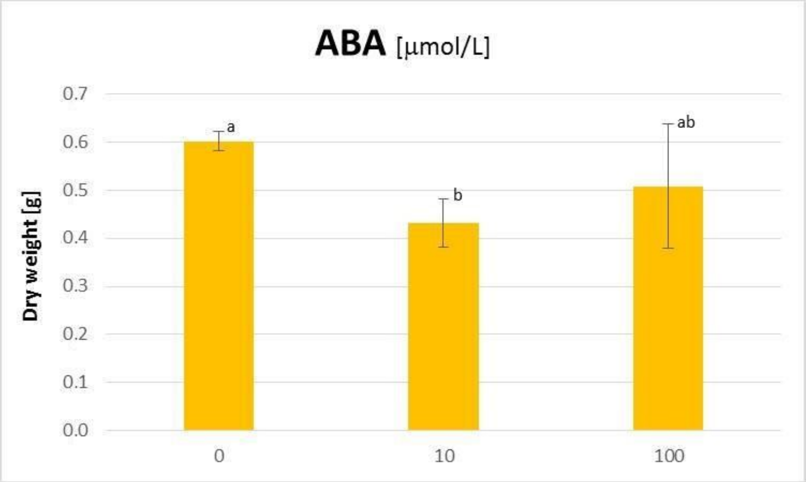

Figure S4. Dry weight of *C. officinalis* hairy root culture after elicitation with abscisic acid.
